# Retraction Note: Impact of different promoters, promoter mutation, and an enhancer on recombinant protein expression in CHO cells

**DOI:** 10.1038/s41598-018-31224-9

**Published:** 2018-09-04

**Authors:** Wen Wang, Yan-long Jia, Yi-chun Li, Chang-qin Jing, Xiao Guo, Xue-fang Shang, Chun-peng Zhao, Tian-yun Wang

**Affiliations:** 1 Department of Biochemistry and Molecular Biology, Xinxiang Medical University, Xinxiang, 453003 Henan China; 2 Pharmacy Collage, Xinxiang Medical University, Xinxiang, 453003 Henan China; 3 School of Life Sciences and Technology, Xinxiang Medical University, Xinxiang, 453003 Henan China

Retraction of: *Scientific Reports* 10.1038/s41598-017-10966-y, published online 05 September 2017

The Authors are retracting this Article.

The Authors were alerted to and confirmed a mix-up of the promoter accession codes for HEF-1α and CHEF-1α promoters. As a result of this error the data and conclusions related to those promoters are swapped and therefore inaccurate.

The Authors recognise this error and apologise for the confusion it may have caused. All authors agree to the retraction.

